# PReS13-SPK-1590: Relevance of cytokine in pediatric inflammatory disease

**DOI:** 10.1186/1546-0096-11-S2-I8

**Published:** 2013-12-05

**Authors:** F De Benedetti

**Affiliations:** 1 Rheumatology, Ospedale Bambino Gesù, Roma, Italy

## 

Cytokines are pleiotropic mediators that play a major role in inducing and orchestrating the inflammatory and the immune response. They play a major role in the response to damage or infections. In some pathological condition abnormal regulation of the production of cytokines leads to pathological events and subsequent damage to target tissues. Dissecting the role of each single mediator in the plethora of cytokines and in the complex networks has been one of the aims of biomedical research that has led to relevant applications in rheumatic diseases. In the presentation we will briefly review the evidence leading to the identification of the role of some cytokines (IL-1, IL-6 and TNF) as key mediators and therapeutic targets in pediatric rheumatic diseases.

## Disclosure of interest

None declared.

